# Solitary Pyomyositis of the Left Rhomboideus Muscle Caused by *Streptococcus anginosus* and *Streptococcus intermedius* in an Immunocompetent Person

**DOI:** 10.1155/2015/321520

**Published:** 2015-06-22

**Authors:** Yasuhiro Tanaka, Kenichi Takaya, Go Yamamoto, Isaku Shinzato, Toshiro Takafuta

**Affiliations:** ^1^Department of Hematology and Clinical Immunology, Nishi-Kobe Medical Center, Kobe, Hyogo 651-2273, Japan; ^2^Department of Orthopedics, Nishi-Kobe Medical Center, Kobe, Hyogo 651-2273, Japan; ^3^Department of Clinical Laboratory, Nishi-Kobe Medical Center, Kobe, Hyogo 651-2273, Japan; ^4^Department of Hematology, National Hospital Organization Kure Medical Center, Kure, Hiroshima 737-0023, Japan

## Abstract

Primary pyomyositis is a bacterial infection of the skeletal muscle commonly affecting children with *Staphylococcus aureus* most often isolated as a pathogen. However, pyomyositis caused by anaerobic bacteria is rare in adults. Here, we report a case of solitary Pyomyositis of the left rhomboideus muscle in an immunocompetent person. A 70-year-old Japanese male presented with high fever and left shoulder pain. His muscle below the lower edge of the left scapula was tender and swollen. His laboratory examinations revealed severe inflammation. Computed tomography showed a solitary low-density area around a contrast enhancement in the left rhomboideus muscle. He was diagnosed as having solitary pyomyositis. Although his symptoms did not improve despite empiric intravenous administration of antibiotics, an incision was performed. *Streptococcus anginosus* and *Streptococcus intermedius* were isolated from the culture of drainage fluid. His symptoms gradually disappeared after the incisional drainage and continuous administration of antibiotics. Pyomyositis did not recur after his discharge. To the best of our knowledge, this is the first report on anaerobic pyomyositis of the shoulder muscle.

## 1. Introduction

Pyomyositis is a purulent infection of the skeletal muscle caused by anaerobic or aerobic bacteria. Pyomyositis usually involves the large muscles located around the pelvic girdle and lower extremities, such as the buttocks and quadriceps, but muscles around the shoulders are rarely involved [1]. The pathogen most commonly isolated from injured skin is* Staphylococcus aureus*; but, in rare cases, pyomyositis could be caused by anaerobic pathogens.

We report here a case of solitary pyomyositis of the left rhomboideus muscle caused by both the anaerobic bacteria* Streptococcus anginosus* and* Streptococcus intermedius*. He was a healthy person, without any underlying disease. To the best of our knowledge, this is the first case report of solitary rhomboideus pyomyositis caused by a mixture of anaerobic pathogens in an immunocompetent person.

## 2. Case Presentation

A 70-year-old Japanese man was admitted to our emergency room because of high fever and left shoulder pain in May, 2013. His medical history was unremarkable and he was taking no medications. He had not undergone any regular medical check-up. About 1 week before admission, he suddenly developed a fever of more than 38 degrees. About 4 days later, he developed pain and swelling of the skin around the left scapula, and he had difficulty in lifting his left upper limb. His symptoms persisted; thus, he was admitted to our hospital. He did not have any recent trauma nor did he travel to any tropical countries.

**Figure 1:**
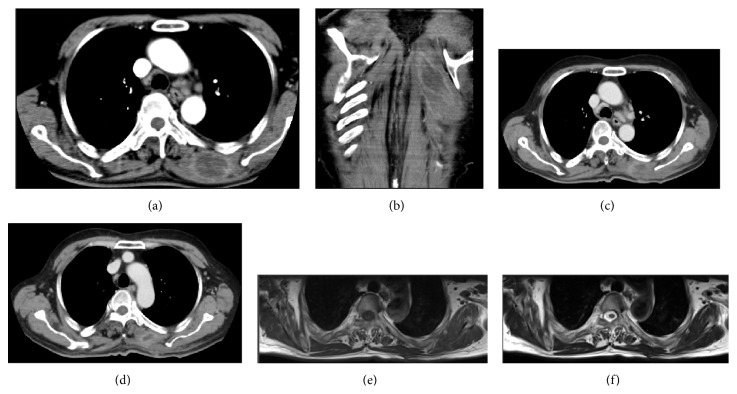
CT and MRI images. (a) Transverse section images of CT on admission showing the swelling of the left rhomboideus muscle and the presence of solitary LDA around a contrast enhancement in this muscle. (b) Coronal section images of CT on admission showing the presence of solitary LDA around a contrast enhancement in the left rhomboideus muscle. (c, d) Transverse section images of CT after drainage showing the residual LDA in the left rhomboideus muscle. (e) T1 weighted MRI images showing no abnormalities in the left rhomboideus muscle. (f) T2 weighted MRI images showing no abnormalities in the left rhomboideus muscle.

On admission, he was in good condition, without abnormalities of vital signs except for a body temperature of 38.4 degrees and a heart rate of 111/min. On physical examination, the flaring and swelling of the skin just below his left scapula were observed. The affected skin area was over 8 cm in its major axis. He also felt tenderness in this area. He had normal heart and respiratory sounds and no abnormalities in the abdominal region. No superficial lymph nodes were palpable, and no rashes or scars were observed in his entire skin. Laboratory examination results indicated severe inflammation: white blood cell count, 17200/*μ*L; C-reactive protein (CRP) level, 13.3 mg/dL. None of the myogenic enzymes showed elevated levels. He did not have diabetes nor did he have human immunodeficiency virus (HIV). Computed tomography (CT) revealed swelling of the left rhomboideus muscle and a solitary low-density area (LDA) around a contrast enhancement in this muscle (Figures 1(a) and 1(b)). No other abnormalities were detected in the thorax or abdomen. From these findings, we suspected the presence of intramuscular abscess; thus, we punctured the affected area. However, the puncture fluid was bloody with no gross pus. After the puncture, we started empiric therapy using intravenous administration of ceftriaxone (CTRX, 2 g b.i.d.). On Day 7 of hospitalization, his condition did not improve and CT showed the enlargement of LDA in the left rhomboideus muscle. No organisms were isolated by the cultures of blood sampled several times. No vegetation was detected by transthoracic echocardiography. On Day 9 of hospitalization, we decided to perform incision and drainage of the left rhomboideus muscle.* S. anginosus* and* S. intermedius* were isolated by the culture of drainage fluid. On oral examination, multiple treated dental caries and periodontitis with pus were found. At that time, he was diagnosed as having solitary pyomyositis of the left rhomboideus muscle. We considered that the source of pyomyositis was periodontitis, although we did not detect a bacterial growth in the pus collected from the dental caries.

After the drainage, we empirically switched the antibiotics to intravenous meropenem (1.5 g t.i.d.) for 14 days; then his symptoms gradually improved. His carious teeth were extracted during meropenem administration. At that time, CT showed residual LDA in the left rhomboideus muscle (Figures 1(c) and 1(d)), and his laboratory examinations showed the persistence of CRP positivity (0.8; normal range, 0 to 0.5 mg/dL). Therefore, we switched to oral amoxicillin (AMPC, 750 mg t.i.d.) on the basis of results of the antimicrobial susceptibility test and continued it for two months. After finishing the AMPC treatment, magnetic resonance imaging (MRI) showed no abnormalities in the left rhomboideus muscle (Figures 1(e) and 1(f)). No relapse occurred more than one year after his discharge from the hospital.

## 3. Discussion

Pyomyositis is a purulent infection of the skeletal muscles that may occur at any age. It involves any muscles, usually the large muscles located around the pelvic girdle and lower extremities, but muscles around the shoulders are rarely involved. Bickels et al. reported the anatomic distribution of primary pyomyositis in 676 patients [1]. They noted that the most frequently involved muscles were those around the quadriceps (26.3%), and the involvement of those around the shoulder including the rhomboideus muscle was found in only 7.9% of these patients. Pyomyositis can usually be a complication in immunocompromised conditions such as HIV and diabetes mellitus [1–3]. Our patient was not immunocompromised, because he was negative for HIV, his HbA1c level was within the normal range, and detailed examinations showed that he had no other diseases or symptoms. Therefore, our case is rare in terms of the pyomyositis occurring around the shoulder muscle in an immunocompetent patient.

The pathogen most commonly associated with pyomyositis is* S. aureus*, which is present in normal skin flora and may cause pyomyositis following a skin injury [1]. However, some anaerobic pathogens, as in our case, could be the cause of pyomyositis. To the best of our knowledge, there were four other case reports of primary pyomyositis caused by* S. anginosus* or* S. intermedius* [3–6] (Table 1). Bickels et al. [1] also noted that there was only one reported case of primary pyomyositis caused by* Streptococcus anginosus-constellatus* and its details were unknown. Unlike our patient, the other four patients had underlying diseases, such as HIV, diabetes, and alcoholism, and their pyomyositis involved the large muscles located around the pelvic girdle and lower extremities, such as the buttocks and quadriceps. This is the first case of solitary rhomboideus pyomyositis in a patient without an underlying disease, that is, an immunocompetent person. The most noteworthy point was that two of the five patients had recent treatments for dental caries or periodontal disease, although the author did not mention the bacterial examinations of samples from the oral infection focus.* S. anginosus *and* S. intermedius* are among the oral flora, and the infection route from the oral cavity to the muscles is not clearly understood. We speculate that the blood stream was the main infection route with transient bacteremia, although the definite pathogen was not isolated by blood culture of samples collected several times from our patient. The exact pathogenetic mechanism was unclear given that the musculature is relatively resistant to infection and that bacteremia rarely leads to muscle infection [7]. It has been proposed that fibronectin binding receptors on muscle cells serve as the path of bacterial entry [8]. In contrast, Takao et al. [9] reported that* S. intermedius* may produce hyaluronidase* in vivo* and* in vitro* and that this enzyme may play a role in host tissue degradation. In the case of pyomyositis caused by* S. intermedius*, hyaluronidase from* S. intermedius* and the fibronectin binding receptor of the host muscle might be necessary for the infection process. Therefore, we should consider checking for dental caries or periodontal disease when we encounter cases of primary pyomyositis caused by anaerobic pathogens.

Our case was unique in terms of long-term administration of antibiotics. Most patients with pyomyositis can be treated successfully with incisional drainage and intravenous administration of antibiotics. Recommendations regarding the optimal duration of the antibiotics have not been determined in adults, but intravenous antibiotics are usually administered for a period of 7 to 10 days, and then oral antibiotics are administered for a total of 5 to 6 weeks often in the case of children [1]. In our patient, residual LDA was found by CT, and CRP positivity was still detected by the laboratory examinations after 14 days of administration of intravenous meropenem, even though both pathogens are sensitive to this drug. Then, oral AMPC was also continued for 2 months. After finishing this oral treatment, no abnormalities in the left rhomboideus muscle were detected by MRI. We are able to explain the exact reason for the prolonged administration of antibiotics, but primary pyomyositis in shoulder regions may require a longer time to resolve than that in the pelvic girdle and lower extremities.

In summary, we described a case of solitary rhomboideus pyomyositis caused by* S. anginosus* and* S. intermedius* in an immunocompetent person.

## Figures and Tables

**Table 1:** Case reports of adult patients with primary pyomyositis caused by *Streptococcus anginosus* and *Streptococcus intermedius*.

Case	Age/sex	Underlying disease	Involved muscle	Pathogen	Drainage	Dental caries
1 Reference [3]	50 M	AIDS	Vastus	*S. anginosus *	Yes	N.D.
2 Reference [4]	34 M	Alcoholism	Quadriceps	*S. intermedius* *E. lentum *	Yes	Yes
3 Reference [5]	31 M	CD	Buttock	*S. anginosus *	No	N.D.
4 Reference [6]	47 M	Alcoholism	Hamstring	*S. intermedius *	No	N.D.
This case	70 M	No	Rhomboideus	*S. anginosus* *S. intermedius *	Yes	Yes

CD: Crohn's disease, AIDS: acquired immunodeficiency syndrome, and N.D.: not described.
